# Social factors associated to binge drinking: a cross-sectional survey among Brazilian students in private high schools

**DOI:** 10.1186/1471-2458-11-201

**Published:** 2011-03-31

**Authors:** Zila M Sanchez, Silvia S Martins, Emerita S Opaleye, Yone G Moura, Danilo P Locatelli, Ana R Noto

**Affiliations:** 1 Brazilian Center of Information on Psychotropic Drugs (CEBRID), Psychobiology Department of the Universidade Federal de São Paulo, São Paulo, Brazil; 2 Department of Mental Health, Johns Hopkins Bloomberg School of Public Health, Baltimore, USA

## Abstract

**Background:**

Binge drinking (BD) seems to be related to health and social complications among adolescents. Considering that knowledge about BD in developing countries is limited and that in Brazil high socioeconomic status is a risk factor for alcohol abuse, this study sheds light about this phenomenon among adolescents from a different cultural background than prior North-American and European studies.

**Methods:**

Brazilian students (n = 2691) selected through a representative, stratified and clustered sampling method were asked to answer a self-report questionnaire. The questionnaire contained questions about patterns of alcohol consumption, religious beliefs, leisure activities, family structure and relationships. Data were analyzed with basic contingency tables with Chi-square tests followed by a decision tree analysis and weighted logistic regression.

**Results:**

Almost thirty-five percent of the students reported recent binge drinking. BD in the past month was positively associated with older age (aOR = 1.5[1.2-1.7]), male gender (aOR = 1.5[1.2-2.0]) going out with friends almost every night (aOR = 33.9[14.2-80.7]), not living with mother (aOR = 2.4[1.3-4.7]), believing in God with little conviction (aOR = 1.6[1.2-2.0]) and rarely talking to parents about anything (aOR = 1.7[1.3-2.2]) or always about drugs (aOR = 1.8[1.3-2.5]). Factors inversely associated with BD were: paying lower monthly tuition fees (aOR = 0.5[0.4-0.9]), living with people who do not get drunk (aOR = 0.6[0.4-0.7]) and frequent engagement in worships (aOR = 0.7[0.5-0.9]).

**Conclusion:**

The habit of BD in adolescents enrolled in private high schools in Brazil is strongly linked to the frequency with which they go out with friends at night. Factors such as religiosity, expressed by trust in God and participation in worship, and being enrolled in a school with cheaper tuition fees were associated with avoidance of BD in this population.

## Background

The term *binge drinking (BD) *has various interpretations and measurements. However, it is most often defined as the consumption of five servings of alcoholic beverages on a single occasion for men and four servings for women [1]. A North American estimate revealed that approximately 90% of the alcohol consumed by underage drinkers is consumed as part of binge drinking episodes [2]. In addition, alcoholic intoxication among adolescents and young adults seems to be related to at least five well-documented complications: 1) traffic accidents, the major cause of death among young individuals between 16 and 20 years old [3]; 2) sexual violence, for both the offender and the victim [4]; 3) memory deficits [5] and the resulting 4) academic impairments [6]; and 5) a higher risk of alcoholism in adulthood [7].

While most European and North American studies emphasize alcohol consumption among adolescents of lower socioeconomic status (SES) [8,9]; according to Brazilian epidemiological studies, high SES is associated with alcohol consumption among Brazilian adolescents [6,10,11]. In an epidemiologic study of 568 high school students aged 14-20 years old in São Carlos (a city in São Paulo state) adolescents with higher SES had higher lifetime prevalence of alcohol use when compared to their low SES counterparts [9]. Carlini-Cotrim et al. [12], compared the risk behaviors of 1675 students between 12 and 18 years old attending public and private schools in the city of São Paulo and found that there was a more pronounced pattern of binge drinking among students of private schools with high tuitions (the wealthiest students). Among these private school students, 25% of the respondents reported at least one episode of binge drinking in the month prior to the research, in contrast with 10% of students in public schools.

In Brazil, wealthy adolescents get enrolled in private schools, since most Brazilian public schools are known to have less educational resources than private ones. This group of students is poorly studied and, the best way to access information from adolescents from higher socio-economic status, is by conducting surveys in private schools. In 2008, around 20% of the students in Sao Paulo were enrolled in private schools [13].

Studies point to family factors as being most prevalent in determining the risk for binge drinking among adolescents. Low parental supervision [14], low quality of family communication, little parental control [15] and a lack of clearly defined behavior rules [16] are associated with alcohol abuse among North American and European adolescents.

Moreover, there seem to be cultural differences in the scope of protection offered by family factors, such as supervision, family structure and quality of relationship with parents. A comparative study of 3984 students from diverse European cities showed that having confidence in one's mother, having a parent at home after school and having parents who care about their children watching too much television were inversely associated with regular use of alcoholic beverage in Rome, Groningen, Newcastle and Bremen, but not in Dublin [17].

Even within family factors, a study among California adolescents showed that the model offered at home to the adolescents would be decisive in the frequent use of alcohol, i.e., parents who drink tend to have teens that replicate this behavior [18]. The strength of the domestic model, coupled with little parental support was also a determinant of early alcohol consumption among students in Taiwan [19]. Furthermore, when this association was investigated among adolescents in Panama, Guatemala and Costa Rica, raised in a Latin culture, the use of drugs/alcohol by a family member seemed not to be associated with episodes of drunkenness among adolescents. In this group, family structure and family interactions was associated to lifetime drunkenness [20]. In addition to family factors, religious factors have been identified as inversely related to alcohol addiction [21], and also to decreased chances of exposure to alcohol among adolescents [22]. Chen et al. [22] in a study among adolescents from Central America identified that higher levels of religious practice (e.g., time allotted for praying and going to church) and religious devotion (e.g., the importance given to attendance at Sunday religious services) were significantly associated with lower odds of initiation of alcohol use. However the actual weight of public and private domains of religiosity is not clear in the decision for non-use or moderate use of alcohol among teenagers [23]. Kendler et al. [24] point to the social aspect of religiosity as possible protector, emphasizing the importance of the frequency of activities within the religious group. Moreover, Wills et al. [25] suggest that religiosity is protective to the experimental use of alcohol only among adolescents who obtained high scores in the private aspects of religiosity, such as faith, prayer and individual emphasis on religion. Moreover, religiosity and family may simultaneously influence the decision of abstinence: religiosity influences family bonding and monitoring and this would influence the youth decision [26].

Furthermore, it is believed that some leisure activities could lead to problematic alcohol consumption, such as parties with friends [27] or sports group practice [28]. However, there are few scientific studies that explore different leisure activities and their association with patterns of alcohol abuse and underage drinking as artistic activities, reading, videogames and internet. As BD can be understood as a leisure activity by adolescents, it is important to verify if there is a pattern of leisure more likely to be associated to BD.

Thus, faced with evidences of possible association of religious factors, family and leisure in alcohol abuse by teenagers, we decided to study the interaction of these factors on the binge drinking behavior of adolescents who attend private high schools (most with high SES which is a major risk factor for BD in Brazil). We chose to study the combined association of these proximal protective and risk factors for BD based on a well known theoretical developmental framework: the Social Development Model [29]. The Social Development Model incorporates propositions of social control, social learning and differential association theories into a developmental framework of both prosocial and antisocial behaviors [30]. According to this model, when adolescents develop bonds with individuals or groups with antisocial beliefs (e.g., drug-using peers), they are more likely to engage in antisocial behaviors; if they develop bonds with individual with prosocial behaviors (e.g., non-drug using peers, high religiosity), they are less likely to engage in antisocial behaviors, and more likely to engage in prosocial behaviors. Also, external constraints such as parental monitoring can affect adolescents socialization experiences [31,32].

As such, the aims of this study are to: 1) Investigate the role of family, leisure and religiosity on BD practices among adolescents in private high schools in São Paulo, Brazil; 2) Examine the role of sociodemographic factors on BD behavior in this population.

## Methods

### 2.1 Sample

The study was designed to select a representative sample of high school students at private schools in São Paulo, Brazil. The city has 578 private high schools and 28 were included in this study. There was some degree of non-participation at the school level, and the standard protocol is to replace these schools with similar schools; 68% of our sampled schools consented; the others were replaced with similar schools in the same stratum. The sample size was set for a maximum relative error of 10% and a confidence interval of 95%. The schools were stratified according to the average incomes of the neighborhoods in which they are located (schools in low- and high-income neighborhoods, in a rate of 2:3). In a second phase, the sample was selected by conglomerates (schools and classes). All students in each selected class were invited to answer the questionnaires. The process generated a final sample of 2691 high school students. The response rate among the students invited to participate was 99.4% (only 16 adolescents refused to participate in the study). The complex survey design took into account the stratum (neighborhoods in which schools are located), the conglomerate (school as primary sampling unit), the expansion weight and the probability of drawing the student who answered the questionnaire.

### 2.2 Data collection

Data collection took place in the classroom, collectively, with no teacher present. A trained team explained the objectives of the project and distributed the questionnaire, which was a self-report paper and pencil instrument with closed questions from an instrument of the World Health Organization [33] and the European School Survey Project on Alcohol and Other Drugs (ESPAD) questionnaire [34], adapted to the Brazilian culture. In addition to questions on the respondent's history and pattern of drinking and other drug use, the instrument collected information on students' SES, religious behavior, leisure activities, and family functioning and structure.

A full re-type of the questionnaires was used to correct all the typing errors. We also included one question on the use of a fictitious drug which, in case of a positive answer (7 cases - 0.2%), excluded the questionnaire from the sample.

### 2.3. Measures

#### Outcome variable

Binge drinking (BD) in the past month, defined as the consumption of five or more servings of alcoholic beverages on the same occasion. A serving was defined as a 5-oz glass of wine, a 12-oz can of beer or a 1.5-oz shot of liquor.

#### Independent variables

Sociodemographic variables (4 questions) selected for this study were: gender, age, and monthly school tuition (obtained directly from schools' principals) and economic scale [35], considering educational level of the head of the household, number of household goods (such as television, DVD players, cars, refrigerators, etc) and number of housekeepers. This scale classifies students from A to E (where A is the highest economic strata). Private school monthly tuition fees were subdivided in quartiles as: US$ 100-190; US$ 191-260; US$ 261-480, US$ 481-1300.

*Leisure activities (7 questions):*The following leisure activities were evaluated according to their frequency in the month that preceded the survey: "going out to bars and parties with friends at night", "hanging out with friends at parks and shopping malls", "playing videogames", "reading books on your own initiative", "using the internet for fun", "participating in artistic activities such as theater, singing and dance, among others" and "participating in volunteer work". All items included responses as *almost every day, at least once a week, one to three times a month*, or *never, except the use of internet that was a dichotomous variable (use x no use in last month)*. *Religiosity (5 questions):*The same pattern of answers was used to investigate the religiosity of the students in the past month through questions on their "voluntary participation in collective prayer", "youth meetings" and "participation in artistic activities within a religious group". We added one question on the "intensity of their belief in God or a higher power", for which possible answers ranged from *none at all *and *very much indeed*. We also included a question on how much the interviewee uses the "ideas of some religion to make decisions", with responses of: *I have no religion; I have a religion but do not use its ideas; I have a religion and sometimes I use its ideas; I have a religion and always use its ideas. Family (20 questions):*There were questions on family structure, such as with whom the respondent lives with (mother, father, stepmother, stepfather, grandparents, siblings) and the marital status of his/her parents. We evaluated parental monitoring and relationship with the respondent through questions on the definition of "rules inside" and "outside" the house; "attention and/or care"; "meals eaten together"; "talks about school", "who they go out with" and "where they go"; "praise" and "conversations lasting at least 10 minutes". In this module, we examined the frequency of each of those behaviors in the last month. The possible answers were as follows: *always, often, sometimes, rarely *and *never*. These parental monitoring and family relationship questions were extracted from the ESPAD questionnaire. Finally, we analyzed alcohol consumption among the people the respondents lived with (alcohol consumption and drunkenness) and the frequency with which they received information on "alcohol" and "other drugs from their parents".

### 2.4 Data analysis

First, we conducted exploratory analyses through basic contingency tables with Chi-square tests and the decision tree (Chaid) with a level of significance of 5%, followed by logistic regression for complex samples with all variables used in the bivariate analysis [36]. Analyses were conducted on data weighted to correct for unequal probabilities of selection into the sample. The outcome variable of interest was binge drinking (BD) in the past month. The independent variables included behaviors related to leisure, religion and family as well as information on drug use. Results are presented via weighted proportions (wgt%), Odds Ratios (ORs), adjusted Odds Ratios (aORs) and p-values.

The complex samples module of the SPSS Version 15 software was used to perform the analyses.

### 2.5 Ethical aspects

The questionnaire did not include any information that could be used to identify the students. Moreover, students were informed of the voluntary nature of the research and the freedom they had to give up at any point or to leave questions unanswered. The study was approved by the Committee of Ethics in Research of the Federal University of Sao Paulo (# 0930/07).

## Results

A total of 2691 students answered the questionnaires. Missing data for the outcome measure (binge drinking) was found in 79 questionnaires (2.9%). Thus, 2612 students were included in the logistic regression and less than 2% had missing data in at least one covariate. 51.9% of the students were female (95%CI 48.9-54.9), 95.5% of higher socioeconomic status (SES A and B; 95%CI 97.4-93.7) and with 16.0 years-old in average (95%CI 15.9-16.1), ranging from 14 to 19 years old. A total of 34.5% of the students reported engaging in binge drinking in the 30 days prior to the research. Table 1 shows the distribution of BD behavior by gender and age. This behavior happens most often, up to 5 times per month, among boys aged 18-19 years.

**Table 1:** Prevalence of past-month binge drinking by gender and age among students in private high schools (n = 2612).

	Gender					Age						
	**Male**		**Female**		**Total**	**14-15**		**16-17**		**18-19**		**Total**

**Binge drinking frequency**	n (wgt%)	95%CI	n (wgt%)	95%CI	n	n (wgt%)	95%CI	n (wgt%)	95%CI	n (wgt%)	95%CI	n

**None**	709 (59.7)	54.8 - 64.4	968 (70.6)	65.9 - 74.8	1677	608 (71.7)	66.7 - 76.1	1005 (63.7)	58.8 - 68.2	64 (47.5)	40.1 - 55.1	1677

**1 time**	138 (10.4)	8.1 - 13.3	139 (10.2)	8.6 - 11.9	277	81 (9.7)	6.9 - 13.4	176 (10.0)	8.1 - 12.4	19 (12.3)	7.5 - 19.4	276

**2 times**	110 (10.1)	8.4 - 12.2	109 (7.1)	5.4 - 9.2	219	67 (7.6)	6.2 - 9.3	135 (8.6)	7.3 - 10.1	16 (13.3)	7.2 - 23.3	218

**3 to 5 times**	150 (12.7)	10.2 - 15.7	107 (7.6)	5.7 - 10.0	257	59 (7.6)	5.5 - 10.4	178 (11.2)	8.6 - 14.5	21 (13.6)	8.6 - 21.0	258

**6 to 9 times**	45 (3.2)	2.0 - 5.0	43 (2.4)	1.5 - 3.8	88	23 (2.0)	1.0 - 4.1	56 (3.1)	2.0 - 4.6	8 (3.8)	1.8 - 7.8	87

**10 or +**	39 (3.9)	2.3 - 6.8	25 (2.2)	1.2 - 3.9	64	9 (1.4)	0.4 - 4.2	47 (3.4)	2.3 - 5.1	8 (9.4)	3.2 - 24.4	64

**Total**	1191 (100)		1391 (100)		2582*	847 (100)		1597 (100)		136 (100)		2580*

Wgt%: percentage weighted

*30 missing data for gender and 32 missing data for age.

Most of the students aged 14-15 years who engaged in BD did so once in the past month (9.7%), but most of the older students (ages 16-19) did so once a week on average (11.5% - 3 to 5 times in the past month). Binge drinking is considered a risky behavior even when it takes place only once per month. Consequently, we chose to analyze the factors associated with BD as a whole, analyzing data from the group that reported at least one episode of BD in the last month as opposed to the group that did not.

After the exploratory bivariate analysis of 36 variables with four categories of answers on average, the chi-square tests detected statistically significant differences for most of the variables tested. To be concise, we present the descriptive bivariate analyses only of the variables that remained in the final logistic regression model in Table 2.

**Table 2:** Bivariate analysis and prevalence of the variables that remained in the final logistic regression model of the students in private high schools (n = 2561).

Variable		Binge				Total	p value
		**No**		**Yes**			

		**n (wgt%)**	**95%CI**	**n (wgt%)**	**95%CI**		

**Gender**	Male	709 (43.9)	40.6 - 47.3	482 (55.9)	50.1 - 61.6	1191	0.001

	Female	968 (56.1)	52.7 - 59.4	423 (44.1)	38.4 - 49.9	1391	

							

**Monthly Tuition**							<0.001

	Up to US$ 190	386 (18.8)	7.4 - 40.0	149 (13.6)	5.1 - 31.5	535	

	US$ 191- US$ 260	631 (42.8)	23.6 - 64.4	347 (41.0)	21.9 - 63.3	978	

	US$ 261- US$ 480	502 (27.5)	12.8 - 49.4	230 (24.4)	11.2 - 45.2	732	

	Over US$ 480	181 (11.0)	3.8 - 27.7	186 (20.9)	7.7 - 45.6	367	

***Family***							

**Living with mother**	No	75 (4.9)	3.4 - 7.1	75 (9.0)	7.0 - 11.5	150	0.002

	Yes	1622 (95.1)	92.9 - 96.6	836 (91.0)	88.5 - 93.0	2458	

							

**10 min talks with parents**							0.043

	Never	42 (2.6)	1.8 - 3.9	25 (2.4)	1.5 - 3.9	67	

	Sometimes/rarely	427 (23.0)	20.1 - 26.3	258 (28.4)	24.4 - 32.6	685	

	Always/often	1226 (74.3)	71.0 - 77.5	625 (69.2)	64.8 - 73.2	1851	

							

**Parents talk about drugs**							0.100

	Never	371 (19.2)	15.8 - 23.1	156 (15.3)	12.7 - 18.2	527	

	Sometimes/rarely	586 (34.4)	32.2 - 36.7	324 (32.7)	26.7 - 39.4	910	

	Always/often	739 (46.4)	42.0 - 50.9	431 (52.0)	44.4 - 59.6	1170	

							

**Someone you live with gets drunk**							<0.001

	No	1480 (89.2)	87.2 - 90.9	713 (81.6)	78.0 - 84.8	2193	

	Yes	186 (10.8)	9.1 - 12.8	174 (18.4)	15.2 - 22.0	360	

***Leisure***							

**You went out with friends at night**							<0.001

	Almost every day	37 (2.5)	1.7 - 3.8	106 (12.9)	9.3 - 17.5	143	

	At least once a week	558 (35.1)	31.6 - 38.8	567 (63.5)	57.5 - 69.1	1125	

	1 to 3 times a month	697 (39.7)	36.0 - 43.6	205 (20.5)	17.1 - 24.3	902	

	Not even once	395 (22.7)	19.4 - 26.2	30 (3.2)	1.7 - 5.8	425	

							

***Religiosity***							

**You trusted in God**							<0.001

	Not at all	125 (7.3)	5.2 - 10.1	103 (10.8)	7.3 - 15.5	228	

	A little/sort of	424 (25.0)	22.5 - 27.7	303 (32.5)	28.4 - 37.0	727	

	A lot/very much	1140 (67.7)	63.3 - 71.9	502 (56.7)	49.2 - 63.9	1642	

							

**Religious services**							<0.001

	Almost every day	93 (6.3)	4.4 - 8.9	23 (2.6)	1.5 - 4.2	116	

	At least once a week	398 (23.3)	19.4 - 27.7	139 (14.3)	10.8 - 18.9	537	

	1 to 3 times a month	308 (17.5)	14.5 - 21.1	201 (22.4)	19.2 - 25.9	509	

	Not even once	886 (52.9)	47.9 - 57.8	540 (60.7)	54.8 - 66.4	1426	

Still in the exploratory phase, the analysis by means of the decision tree (CHAID) identified the frequency of "going out to bars and parties with friends at night" as the factor most strongly associated with binge drinking (χ^2 ^= 419.3; df = 3; p > 0.001). This effect is so pronounced that, in addition to being the first knot in the tree, there was no other knot for those who reported the practice of BD; that is, if going out with friends at night is considered, no other variable can further separate those who engaged in BD from those who did not.

When complex samples logistic regression was performed, the final model did not reveal as many differences as expected in the exploratory analysis. BD was significantly associated with the following sociodemographic variables: male gender (OR = 1.53[1.16-2.01]; p = 0.004), older age (increment of 1.46 of OR for each year; p < 0.001) and schools charging higher tuition, as presented in Table 3.

**Table 3:** Results of the logistic regression for factors associated with the practice of binge drinking in the month prior to the research among students in private high schools (n = 2561).

Variable		cOR[95%CI]	aOR[95%CI]	p value
***Sociodemographic***				

**Age**		1.36[1.20-1.52]	1.46[1.23-1.73]	< 0.001

**Gender**	Male	1.62[1.26-2.08]	1.53[1.16-2.01]	0.004

	Female			reference

**Monthly Tuition**	US$ 100- US$190	0.38[0.29-0.46]	0.59[0.39-0.91]	0.018

	US$ 191- US$ 260	0.50[0.40-0.64]	0.59[0.42-0.83]	0.004

	US$ 261- US$ 480	0.47[0.32-0.68]	0.53[0.33-0.85]	0.011

	US$ 481 -US$ 1300			reference

***Family***				

**Living with mother**	No	1.91[1.31-2.78]	2.43[1.26-4.07]	0.010

	Yes			reference

**10 min talks with parents**	Never	1.32[1.06-1.66]	1.68[1.28-2.21]	0.003

	Sometimes/rarely	0.99[0.55-1.81]	1.51[0.70-3.28]	0.280

	Always/often			reference

**Parents talk about drugs**	Always/often	1.41[1.16-1.80]	1.80[1.29-2.52]	< 0.001

	Sometimes/rarely	1.19[0.88-1.61]	1.24 [0.92-1.68]	0.149

	Never			reference

**Someone you live with gets drunk**	No	0.54[0.40-0.73]	0.56[0.44-0.71]	< 0.001

	Yes			reference

***Leisure***				

**You went out with friends at night**	Almost every day	36.23[15.01-87.44]	33.88[14.22-80.70]	< 0.001

	At least once a week	12.77[7.30-22.32]	12.09[6.78-21.54]	< 0.001

	1 to 3 times a month	3.63[1.86-7.10]	3.39[1.71-6.72]	0.001

	Not even once			reference

***Religiosity***				

**You trusted in God**	Not at all	1.78[1.27-2.47]	1.35[0.88-2.07]	0.159

	A little/sort of	1.55[1.28-1.88]	1.58[1.22-2.03]	0.001

	A lot/very much			reference

**Participation in religious services**				

	Almost every day	0.36[0.21-0.60]	0.50[0.27-0.91]	0.026

	At least once a week	0.53[0.44-0.65]	0.69[0.53-0.88]	0.005

	1 to 3 times a month	1.11[0.87-1.42]	1.22[0.91-1.64]	0.166

	Not even once			reference

aOR = adjusted odds ratio (final model)

cOR = crude odds ratio

The most significant of the leisure activities evaluated was the amount of time the students spent socializing with their friends outside their homes at night. Going out with friends almost every night to bars, shows and parties increased the odds of BD by up to 33.88 times ([14.22-80.70],p < 0.001). Even at lower frequencies (one to three times in the month versus at least once a week), this variable posed a potential risk for binge drinking, with odds between 3.39([1.71-6.72], p = 0.001) and 12.09 times higher ([6.78-21.54], p < 0.001) than among those who did not go out even once in the past month.

Regarding family structure, not living with one's mother (OR = 2.43, [1.26-4.07], p = 0.010) was the only variable that was significantly associated with BD, irrespective of the parents' marital status or of one parent being deceased (either father or mother).

When parental monitoring was evaluated, having parents who never talk to the respondents for at least 10 minutes a day was positively associated with BD (OR = 1.68[1.28-2.21], p = 0.003) when compared to those with parents who always did that. Also, having parents who always talked about drugs was positively associated with BD (OR = 1.80, [1.29-2.52], p < 0.001).

Variables such as the definition of behavioral rules, attention, praise from parents, parents' knowledge about where their children are and with whom, having meals together, talking about alcohol and about school and whether the children receive an allowance were not significant in the model. However, if nobody the student lives with gets drunk, the chances of BD are reduced by 44% (p < 0.001).

From the religiosity module, the only two variables that remained in the model were the frequency with which the adolescents attended collective prayer (masses, services) and their level of trust in God. Individuals who attended group prayer at least once a week and almost every day had 31 and 50% less chance of being involved in binge drinking ([0.53-0.88], p = 0.005 and [0.27-0.91], p = 0.026, respectively). Therefore, when "trust in God a lot or very much" was defined as the reference group, trusting a little or moderately was positively associated with the practice of binge drinking (OR = 1.58, [1.22-2.03], p = 0.001).

## Discussion

The main findings of this study can be summarized as follows: 1) Going out with friends frequently at night was the strongest factor associated with BD; 2) BD was also significantly associated with older age, male gender, not living with mother, rarely talking to the parents or having meals with them and having little trust in God. 3) On the other hand, being enrolled in a school with a cheaper tuition, attending group prayer meetings at least once a week and not living with someone who gets drunk were inversely associated with BD.

The practice of BD by students in private high schools in the city of São Paulo had a much higher prevalence than the Brazilian national prevalence for this behavior among adolescents (16% in last year; [37] and than the students' American peers (29% [38]). This can be partially explained by the fact that these students are mainly from the highest SES in the Brazilian population [10], even though monthly tuition costs varied widely from US$ 100 to US$ 1500. Even though the most recent North American data show gender equivalence of this practice among high school students [38], this practice was more strongly associated with male gender in our study. This is similar to what occurs among urban adolescents in China, an emerging economy as Brazil [39] and among European adolescents [40].

The students who attended schools charging lower monthly tuition appeared to be more protected against the practice of binge drinking, suggesting that this behavior is linked with family income and SES. Previous Brazilian studies had already associated higher consumption of alcohol and other drugs with higher family income among high school students [11] as well as college students [41]. This lower rate of BD in schools with cheaper tuitions may occur by the divergent anti-drug policy in the schools or even because differences in pocket money students receive from their parents.

### Leisure activities

The most substantial findings of this study point to the relevance of the role of friends in binge drinking, which suggests the influence of social learning and social control, as proposed by the Social Development Model [31]. When family, religious and leisure variables were taken into account, the strongest factor associated with binge drinking behavior was the frequency of going out to parties and bars with friends at night. Attention should be paid to this issue given the high crude and adjusted odds ratio and its prominence as the first knot in the decision tree. However, since this is a cross-sectional survey, it is not possible to establish any causal link. It is not possible to establish what comes first: the exposure to binge drinking or the intent to practice it. According to our findings, there is evidence of an association of binge drinking with the presence of friends, the party environment and physical distance from the parents. The more an adolescent socializes away from home at night in places where he can drink with friends, greater his chance of engaging in binge drinking.

Previous studies of college students also highlighted the strong influences of the social context and friends regarding this specific behavior, once more suggesting that the Social Developmental Model plays a central role in the drinking behavior of young adults also. Going out to parties and bars at night certainly exposes the adolescent to a risky environment, and college binge drinking and access to alcohol is very easy, as observed by Weitzman et al [42]. In addition, Beets et al [27] found that binge drinking is strongly associated with parties, especially on weekends. However, it is believed that the facilitated access to alcohol does not happen only at night in the so-called "parties". In Brazil, it is widely recognized that adolescents can easily buy alcohol even though the law prohibits its sale to individuals under 18, as Romano et al [43] already described. The easy access, along with low prices (a bottle of *cachaça *[fermented sugarcane liquor] or beer can be bought for a little more than US$1), imitation of the behavior of friends and physical distance from the parents seem to be a high-risk combination for binge drinking.

Other leisure activities beside those related to socializing outside the home are not associated with binge drinking. Interestingly, in the context that many preventive programs for alcohol and other drugs use among adolescents are focused on improving the use of free time it is important to notice that there was no evidence that leisure sports activities in these adolescents' free time, artistic activities, internet use or reading were associated with binge drinking.

### Family

In the context of physical distance from the parents and almost daily socialization with friends on outings or at parties, it was believed that the variables regarding parental supervision would present significance in the final model, as demonstrated by Mistry et al [18] for both boys and girls engaged in drinking and smoking. Nevertheless, our data showed that only the students who reported that talked to their parents for 10 minutes or more per day seemed to be less exposed to BD. Guilamo-Ramos et al. [15] characterized the family variables that influence BD as little dialogue, supervision and control. We did not detect associations with direct ratings of supervision and control, but associations were found with the possible protective effect of conversation during the day. On the other hand, we observed a positive association with BD when parents and children regularly talk about drugs: adolescents who reported always or often talking to their parents about drugs were almost twice as likely to have engaged in a binge. A justification for this finding might be that those parents are already suspicious of their children's risky alcohol consumption and are trying to reduce their chances of using illegal drugs, maybe believing that using "drugs" is more serious than getting drunk. Another hypothesis that was not tested is that teenagers who are binge drinking are also using other drugs and their parents are more concerned about the consumption of drugs other than alcohol than about the practice of binge. Or, perhaps this is simply the result of a measurement issue, and parents are not necessarily distinguishing between alcohol and other drugs when they talk to their kids.

Considering the parents-child discussion issue, Austin et al (2000)[44] found that that parental discussion about alcohol can worsen perceptions about the media message. Their study shows that positive parental mediation of television advertising seems to increase risk for alcohol use while negative parental mediation decreases the risk. This finding points to the need of a future examination of the content of the 10 minute talks that is protecting adolescents from binge drinking.

Family structure was another aspect that presented relative disagreement with published data. In this model, parents' marital status did not present itself as a protective or risk factor for binge drinking, as opposed to the North-American findings of Miller et al., [45] according to which living with both parents is a protective factor against alcohol intoxication and of Ledoux et al. [14], who confirmed that adolescents from French and UK who lived in non intact families (divorced or dead parents) were more likely to binge drinking in the past 30 days. The presence of the mother seems to be the strongest associated factor of the variables of family structure. This finding may be explained based on the lifestyle of youths who do not live with their mothers, which could be less orderly or disciplined. The presence of the mother might provide more control over the actions of the youth. This study did not investigate the quality of the relationship between the adolescents and their parents or how long they have lived together. However, the maternal role seems to be a potent inhibitor across different countries and cultures. A major task of adolescence is seeking autonomy from parents while increasing reliance on peers. More secure attachment styles and maternal-adolescent relationship quality in this period had a positive effect in the influence of peer substance use [46]. Moreover McAdler et al. [17] suggest that the involvement of the adolescent with his/her mother is more influential than her physical presence regarding the adolescent's heavy use of alcohol. The closer they are to their mothers, the less binge drinking adolescents between 14 and 15 years old engage in. However, these findings are not in line with those of Johnson et al. [47], who observed that deeper affection for the mother and better communication between parents and children were associated with a higher intake of alcohol. The authors believe that mothers do not often see the use of alcohol as a problem, mainly as a result of their trust in their children. However it can be also interpreted that sometimes children can evaluate the permissiveness of their mothers as an evidence of deeper affection. In addition, in this European study, McAdler et al. [17] found that living with both parents was not associated with alcohol consumption, but to the use of other drugs. As detected in previous studies carried out among adolescents [11], alcohol use and dependence of the parents are directly related to their children's abusive alcohol use. In this representative sample, living with someone who usually gets drunk practically doubled the odds for the adolescent to engage in BD. Hung et al. [19] also found that adolescents who have parents who both used alcohol and less parental support were significant predictors of early onset of alcohol use.

For Kuntsche et al. [40] it happens because adolescents who are more likely to engage in BD tend to underestimate the problem due to their family history of drunkenness. As family factors, especially parents drinking, influence children's initiation of alcohol use, it is important to teach parents about alcohol effects on children and to emphasize their role in prevention. Moreover, Arria et al. [48] emphasize the importance of awareness among parents on their role in determining their rules on alcohol consumption in adolescence, whereas family influence is quite strong this time and loses sustainability at the stage where the children go to University.

The protective effect of the model received at home may also indicate interventions emphasizing controlled alcohol consumption by parents. Thus, a prevention program could include brief intervention for parents who abuse alcohol triggering drunkenness. In this sense, Mistry et al. [18], studying the process of resilience among adolescent, found that those who are considered drinkers generally have models of this behavior at home. In fact, not only the behavior for alcohol use is a reflection of domestic experience, but also practice of physical activities and balanced meals, with fruits and vegetables. Besides, authors suggest that any program of health promotion should be founded on family and ethnically targeted, considering the peculiarities of cultural backgrounds.

### Religiousness

Finally, the analysis of the five religiosity variables showed the association of two of them with the practice of binge drinking. As observed before by Sinha et al. [49] adolescents who attended religious services (group prayer) most often were the least exposed to risk behaviors, as is the case of heavy alcohol use. Our findings show relevance of almost daily or at least weekly participation in group prayer and a high trust in God, but no association with the frequency of attendance at artistic activities within the religious group (such as theater classes, dance and music classes inside a church and with religious background) or youth group meetings. Kliewer and Murrelle [20] have hypothesized that the more an adolescent believes in God, the more protected he will be against the use of legal and illegal drugs. Indeed, church services are a way to occupy the time of youth in alternative activities besides binge drinking, but also, in this religious activity they should learn moral concepts that discourage alcohol abuse and possible enlarge their trust in God. In this sense, Stylianou [50] proposed a theory suggesting that the perception of immorality and personal responsibility on physical self-destruction that religions bring to their members controls the attitudes of these individuals when faced with opportunities for drug use.

Notably we found an inverse association between BD and a factor of intrinsic religiosity (trust in God) and one factor of organized religiosity (participation in group prayer/religious services) that has already been explored in studies presented by Koenig et al. [21], especially among North-Americans. As this is a cross-sectional survey, we cannot establish what came first: the attendance at cults that developed the trust in God or because the adolescent trusts in God s/he seek to attend a group prayer weekly? It is important to emphasize that the use of religious belief in life decisions was not associated with BD in our sample, suggesting that perhaps more important than what is said in the church is the network of non-drinking friends that adolescents is able to contact in these places, favoring a less risky lifestyle.

We should bear in mind, though, that not all religions in Brazil have a clear position about alcohol use, and therefore their followers might see them as permissive, which would not curb their abusive use [51]. As Catholics take alcohol on their sacrament and Mormons forbid alcohol use, we would find different association among drinking behavior according to religious affiliation [52]. However, on this study we have no information about religious affiliation and it should be seen as a limitation.

### Limitations

Other important limitations should be pointed out. Since it is a cross-sectional survey, the factors analyzed might present associations with the practice of binge drinking, but it is not possible for us to establish causal relations. Moreover, the data collection in the classroom might exclude students who are often absent from class or those who developed serious alcohol-related problems and dropped out of school. Additionally, due to the fact that a self-report questionnaire was used, the answers are only reports of consumption, and the questions are subject to interpretation by the participants. The adolescent respondents might be underreporting their drug use [53]. Moreover, we did not have data from students in public high schools in São Paulo, so data is not generalizable to the general adolescent Sao Paulo population and the BD pattern might be different in private schools in other Brazilian cities and regions. On the other hand, the strength of this study is the rarity of the sample. Carlini-Cotrim et al. [12] had already mentioned the difficult of accessing private schools in Sao Paulo, a fact that was actually noticed by our group, but that was circumvented through various telephone contacts and visits to school principals.

## Conclusion

Considering that knowledge about BD in developing countries is limited, as most research has been conducted among North-American college students and other particular groups of young adults, this study sheds light about this phenomenon among younger students from a different cultural background. Besides adding to scientific knowledge on BD, this study provides a step forward in identifying the groups that prevention programs should target.

The practice of BD among wealthy high school students in São Paulo seems to be strongly associated with the time they spend at parties, shows and bars at night, consistent with the Social Developmental Model as the main factor associated with this practice was the exposure to a party environment with friends at night. In this sense, their friends are playing a crucial role in this behavior. Future studies should investigate what happens in those environments from an ethnographic perspective and determine what protects some youths from engaging in the practice of binge drinking. Furthermore, participation in group prayer in a religious environment seems to be alternatives to going out with friends and, as such an alternative to be exposed to alcohol and binge drinking. Finally, a component of parental monitoring and family structure were also associated with binge drinking, such as the presence of the mother figure and talks lasting at least 10 minutes. This shows the importance of dialogue between parents and children, which could be stimulated by school projects for prevention of binge drinking. Since the present study suggests that parties with friend are more important for the binge drinking behavior than the protection that comes from religiosity or family support, one can imagine that a model of positive peer pressure could be tested as a prevention program for this specific behavior.

## Competing interests

The authors declare that they have no competing interests.

## Authors' contributions

ZMS: wrote the manuscript and did the analyses; SSM: supervised the analysis and the paper writing process; ESO, DPL and YGM: literature revision, data discussion and data collection; ARN: conceptualized the manuscript and was the project coordinator. All authors read and approved the final manuscript.

## Pre-publication history

The pre-publication history for this paper can be accessed here:

http://www.biomedcentral.com/1471-2458/11/201/prepub
